# Activation of Type III Interferon Genes by Pathogenic Bacteria in Infected Epithelial Cells and Mouse Placenta

**DOI:** 10.1371/journal.pone.0039080

**Published:** 2012-06-14

**Authors:** Hélène Bierne, Laetitia Travier, Tanel Mahlakõiv, Ludovic Tailleux, Agathe Subtil, Alice Lebreton, Anupam Paliwal, Brigitte Gicquel, Peter Staeheli, Marc Lecuit, Pascale Cossart

**Affiliations:** 1 Institut Pasteur, Unité des Interactions Bactéries Cellules, Paris, France; 2 Inserm, U604, Paris, France; 3 INRA, USC2020, Paris, France; 4 Institut Pasteur, Groupe Microorganismes et Barrière de l’hôte, Paris, France; 5 Inserm, avenir U604, Paris, France; 6 Department of Virology, University of Freiburg, Freiburg, Germany; 7 Spemann Graduate School of Biology and Medicine (SGBM), University of Freiburg, Freiburg, Germany; 8 Institut Pasteur, Unité de Génétique Mycobactérienne, Paris, France; 9 Institut Pasteur, Unité des Biologie des Interactions Cellulaires, Paris, France; 10 Université Paris Descartes, Hôpital Necker-Enfants malades, Service des Maladies Infectieuses et Tropicales, Paris, France; Universite de la Mediterranee, France

## Abstract

Bacterial infections trigger the expression of type I and II interferon genes but little is known about their effect on type III interferon (IFN-λ) genes, whose products play important roles in epithelial innate immunity against viruses. Here, we studied the expression of IFN-λ genes in cultured human epithelial cells infected with different pathogenic bacteria and in the mouse placenta infected with *Listeria monocytogenes*. We first showed that in intestinal LoVo cells, induction of IFN-λ genes by *L. monocytogenes* required bacterial entry and increased further during the bacterial intracellular phase of infection. Other Gram-positive bacteria, *Staphylococcus aureus, Staphylococcus epidermidis* and *Enterococcus faecalis,* also induced IFN-λ genes when internalized by LoVo cells. In contrast, Gram-negative bacteria *Salmonella enterica* serovar Typhimurium, *Shigella flexneri* and *Chlamydia trachomatis* did not substantially induce IFN-λ. We also found that IFN-λ genes were up-regulated in A549 lung epithelial cells infected with *Mycobacterium tuberculosis* and in HepG2 hepatocytes and BeWo trophoblastic cells infected with *L. monocytogenes*. In a humanized mouse line permissive to fetoplacental listeriosis, *IFN-λ2/λ3* mRNA levels were enhanced in placentas infected with *L. monocytogenes*. In addition, the feto-placental tissue was responsive to IFN-λ2. Together, these results suggest that IFN-λ may be an important modulator of the immune response to Gram-positive intracellular bacteria in epithelial tissues.

## Introduction

Type III interferons (IFN-III or IFN-λs) are recently described cytokines involved in antiviral responses (for reviews [1]–[3]). Early studies had suggested that they were functionally redundant with type I interferons (IFN-I, *e.g.* IFN-α, IFN-β), but several reports indicate that IFN-λs also have specific functions, particularly in epithelial tissues [4]–[6]. The human genome harbours three functional IFN-III genes: *IL-29* (*IFN-λ1*), *IL-28A* (*IFN-λ2*) and *IL-28B* (*IFN-λ3*). These genes share common regulatory elements with IFN-I genes in their promoter region, with predicted binding sites for transcriptional factors NF-κB (nuclear factor κB), IFN regulatory factors (IRFs, especially IRF3 and IRF7) and AP1 (a dimeric transcription factor containing members of the JUN, FOS, ATF and MAF protein families). Hence various cell types can co-produce IFN-I and IFN-III in response to viral infection [3], [7]–[9]. However, the mechanisms of regulation of IFN-I and IFN-III encoding genes are not strictly identical. In particular, recent reports support the fact that IFN-III, in contrast to IFN-I genes, can be activated by the NF-κB pathway independently of IRFs [7], [10].

Another difference between the IFN-I and IFN-III pathways is the use of distinct receptors. IFN-λs specifically interact with a heterodimeric receptor composed of two chains, a specific ligand-binding chain IFN-λR1 (or IL-28Rα) and the IL-10R2 (or IL-10Rβ) chain. In contrast, IFN-I are ligands of the IFNAR receptor. Both receptors induce the JAK/STAT signaling pathway, leading to transcriptional activation of similar sets of IFN-responsive genes [11], albeit with different kinetics [12]. Even though IFN-I and IFN-III display similar biological properties, such as antiviral and antitumor activities [1]–[3], [13], [14], their physiological roles are distinct because of the distribution of their receptors in tissues. In fact, unlike IFNAR, the IFN-λ receptor is not detectably expressed in hematopoietic cells, fibroblasts or endothelial cells, while it is primarily expressed in epithelial cells and specific subsets of immune cells, therefore acting predominantly at mucosal surfaces [4], [5], [15], [16]. An IFN-λ response was also reported in human liver, where it seems to play an important role in chronic hepatitis C [17]. This particular cell tropism explains why IFN-III is increasingly recognized as a key mediator of immunity in specific niches.

IFN genes are expressed following recognition of microbial-associated molecular patterns (MAMPs) by pattern recognition receptors (PRRs), acting as sensors for microbial products at the cell surface or in endosomal or cytosolic compartments [18]. IFN-III genes are activated in monocyte-derived dendritic cells (DCs) following stimulation with bacterial components, such as lipopolysaccharide (LPS) and other TLR4 and TLR9 ligands [2], [19], [20]. However, whereas the role of IFN-I in bacterial infections has been extensively investigated [18], [21], [22], the role of IFN-III has not been explored, except in two recent studies. Pietilä *et al.* have described that the transcription of *IFN-λ1* and *IFN-λ2/λ3* mRNAs increases in response to infection of human DCs with the Gram-negative pathogen *S. enterica sv* Typhimurium [20]. We have shown that the Gram-positive bacterium *L. monocytogenes* stimulates *IFN-λ2* transcription during infection of epithelial cells and that this pathogen is able to tightly control the expression of the downstream responsive genes at chromatin level by hijacking a chromatin-silencing complex [23], [24]. This modulation by a bacterium of IFN-III responses supports the hypothesis that IFN-III contributes to immunity of epithelia against invading bacteria.

So far, no bacterial species other than *L. monocytogenes* has been examined for the induction of IFN-λ genes in epithelial cells. Because a wide range of bacterial pathogens colonize epithelia, it was of interest to compare the ability of several bacterial pathogens to induce these genes in this cell type. Here, we further detail *L.* *monocytogenes-*mediated stimulation of *IFN-λ1* and *IFN-λ2* in Lovo intestinal cells, and we show that there are striking differences between bacterial species in the induction of IFN-λ genes in epithelial cells. In addition, we provide evidence that the placenta is a tissue in which IFN-III genes are induced upon bacterial infection and where IFN-III responses can be elicited.

## Materials and Methods

### Bacteria and Human Cells

Bacteria used in this study are listed in Table 1. All bacterial strains (except *Salmonella*, *Chlamydia* and *Mycobacterium*) were grown in brain-heart infusion (BHI) at 37°C, and for strains expressing *inlA*, in the presence of chloramphenicol, 7 µg/ml. *Salmonella* was grown in Luria-Bertani (LB) media (Difco) at 37°C. *GFP*-expressing *M. tuberculosis* was grown from a frozen stock to mid-log phase in 7H9 broth supplemented with albumin-dextrose-catalase (ADC, Difco). *C. trachomatis* serovar L2 were grown on HeLa cells, collected, as described [25], and stored at -80°C until use. Human cell lines used are as follows: colon carcinoma epithelial LoVo cells (ATCC CCL-229), in which *L. monocytogenes* is efficiently internalized by the InlA and InlB pathways [26], placental BeWo cells (ATCC CCL-98), HepG2 hepatocytes (ATCC HB-8065), lung alveolar basal epithelial A549 cells (ATCC CCL-185), carcinoma epithelial Caco-2 cells (ATCC HTB-37), HEK293 embryonic kidney cells (CRL-1573) and monocyte THP-1 (ATCC TIB-202). All cell lines were grown under standard cell-culture conditions following ATCC recommendations.

**Table 1:** List of bacterial strains used in this study and their effect on IFN-III genes in epithelial cells.

Bacterial species	Strain	Reference ^(a)^	Effect on IFN-III ^(b)^	Human cell line	Reference ^(c)^
*Listeria monocytogenes*	EGDe	ATCC-BAA-679 BUG1600	++	LoVo	[23] and this work
			++	HepG2	This work
			++	BeWo	This work
			++	JEG-3	[23]
			++	Caco-2	This work **^(d)^**
			–	HEK293	This work **^(d)^**
			–	THP-1	This work **^(d)^**
*Listeria monocytogenes*	EGDe Δ*inlA*	BUG1454 [57]	–	LoVo	This work
*Listeria monocytogenes*	EGDe Δ*inlB*	BUG1455 [57]	–	LoVo	This work
*Listeria monocytogenes*	EGDe Δ*hly*	BUG2133 [58]	+/−	LoVo	This work
*Listeria innocua*	*Clip11262*	ATCC BAA-680 BUG499	–	LoVo	This work
*Listeria innocua* (*inlA)*	BUG1496	BUG1496 [31]	+	LoVo	This work
*Enterococcus faecalis*	OG1X	BUG1491 [31]	–	LoVo	This work
*Enterococcus faecalis (inlA)*	BUG1497	[31]	+	LoVo	This work
*Staphylococcus epidermidis*	BUG1477	[31]	–	LoVo	This work
*Staphylococcus epidermidis* (*inlA)*	BUG1498	[31]	+	LoVo	This work
*Staphylococcus aureus*	SH1000	[59]	++	LoVo	This work
*Salmonella enterica* serovar Typhimurium	SL1344	ATCC SL1344	–	LoVo	This work
*Shigella flexneri*	M90T	[60]	–	LoVo	This work
*Chlamydia trachomatis* serovar L2	VR902B	ATCC VR902B	+/−	LoVo	This work
*Mycobacterium tuberculosis (GFP)*	H37Rv	[27]	++	A549	This work

(a) Reference of the strain.

(b) Effect on the expression of IFN-III. “++”: high induction; “+”: induction; “+/−”: weak induction; “–”: no induction.

(c) Reference for the observed effect on IFN-III genes.

(d) Data not shown.

### Bacterial Infections in Human Cells

Cells grown to 70–90% confluency were infected with different bacteria at indicated multiplicity of infection (MOI) and following standard protocols. Invasion efficiency was quantified by either measuring bacterial loads after gentamicin treatment or by flow cytometry. ***(i)*** *Gentamicin assays.* Briefly, bacteria grown to the early stationary phase and washed twice in PBS were diluted in culture medium to achieve a multiplicity of infection (MOI) of 10, 20, 25 or 100. Inocula were used to infect cells for the indicated time, with extracellular bacteria killed by adding gentamicin 25 or 50 µg/ml 1 h post-infection. The number of cells per well was determined with a cell counter (Countess, Invitrogen) following cell detachment with Trypsin/EDTA. Intracellular bacteria were liberated by the addition of 0.2% Triton X-100 in PBS and quantified by plating serial dilutions of cell lysates on BHI or LB agar plates and numbering colony-forming units (CFU). Experiments were performed in duplicates or triplicates and reproduced three to five times. ***(ii)*** *Mycobacterium infection*. Before infection, bacteria were washed twice and resuspended in 1 ml PBS. Clumps were disassociated by passages through a needle, followed by 5 min of sedimentation. The density of bacteria in the supernatant was checked at OD_600_ and correlated to the numeration of the aliquot to allow 10 or 25 bacteria per cell. After 18 h of infection, half of the cells were lysed for RNA extraction with RLT buffer from RNeasy Mini Kit (Qiagen), and half were fixed in paraformaldehyde and analyzed by flow cytometry to quantify the percentage of infected cells (GFP-positive), using a FACS flow cytometer (FACSCalibur, Becton Dickinson), as described previously [27]. Experiments were performed in duplicates and reproduced four times. **(** ***iii*** **)** *Chlamydia infections*. Cells were inoculated with *Chlamydia* at a MOI of 0.5 to 1. After 90 min at 37°C, the culture medium was changed and the plates returned to 37°C for 24 h before RNA extraction. Cells were fixed in 70% ethanol and used to quantify the percentage of infected cells by flow cytometry, using FITC-coupled anti-*Chlamydia* antibody (Argene #12–114). Experiments were performed in quadruplicates and reproduced once.

### RNA Isolation, Reverse Transcription and Quantitative Real Time PCR (qRT-PCR)

RNA from infected or uninfected cells was extracted using RNeasy Mini Kit (Qiagen). Genomic DNA was removed by treatment with TURBO DNA-free™ kit (Ambion) and RNA concentration and purity was assessed using NanoDrop spectrophotometer (Thermo Scientific). First strand cDNA was synthesized from 1 to 2 µg total RNA by using the RT^2^ first strand kit (SABioSciences, Qiagen) and quantitative PCR was performed with RT^2^ qPCR Primer Assay using the manufacturer’s protocol (SABioSciences, Qiagen) and the recommended two-step cycling program, on a MyIQ device (Bio-Rad). Each reaction was performed in triplicate. All human and mouse qRT-PCR primers were pre-designed, validated RT^2^ qPCR primer pairs (SABioSciences, Qiagen; see below). Relative gene expression was normalized to *GAPDH* or *YWHAZ* reference gene transcription and calculated with the ΔΔC_T_ method. Statistical analysis was performed on triplicates from two to six independent experiments using Student’s two-tailed T-test. A p-value *p*<0.05 was considered statistically significant. Primers used for qRT-PCR were pre-designed, validated RT^2^ qPCR primer pairs (SABioSciences, Qiagen) as follows: for human genes, IL28A (IFN-λ2, PPH05847A), IL29 (IFN-λ1, PPH05849A), IFNB1 (IFN-β, PPH00384E), IFNG (IFN-γ, PPH00380B), GAPDH (PPH00150E), YWHAZ (PPH01017A), IL-8 (PPH00568A), and for mouse genes IL28A (IFN-λ2, PPM34810A), IL28B (IFN-λ3, PPM34810A), IFIT1 (PPM05530E), Mx1 (PPM05520A), Mx2 (PPM05503A), IGF2 (PPM03655A), GAPDH (PPM02946E) and YWHAZ (PPM03697A).

### ELISA Assays

Cytokines in the supernatant of epithelial cells were quantified with enzyme-linked immunosorbent assay (ELISA) according to the manufacturer’s instructions, with the following references: IFN-λ1/λ3 and IFN-λ2 (Duoset ELISA kit, DY1598B and DY1587, R&D Systems), IFN-β (VeriKine ELISAs, #41410-1A, PBL Biomedical Laboratories, Piscataway, NJ, USA) and IL-8 (IL-8 Ready-SET-Go!, #88–8086, eBioscience).

### *L. monocytogenes* Infections, Quantification of IFN Transcripts and Mx1 Staining in Mouse Placenta

#### Ethics statement

All animal experiments in the Pasteur Institute were performed in accordance with protocols approved by the animal Experimentation Ethics Committee of the Pasteur Institute (permit #03–49). All animal experiments in the University of Freiburg were performed in compliance with the German animal protection law (TierSchG) and were approved by the local animal welfare committees. The animals were housed and handled in accordance with good animal practice as defined by FELASA (www.felasa.eu/guidelines.php) and the national animal welfare body GV-SOLAS (www.gv-solas.de/index.html). Pregnant humanized E16P mEcad (E16P^+/+^) [28] and B6.A2G-Mx1-IFNAR1^0/0^ [4]–[6] mice were used. ***(i)*** *Murine fetoplacental listeriosis experiments*. 4×10^4^ bacteria in PBS were injected intravenously in the tail vein of 10 week-old pregnant homozygous knock-in mice expressing human E-cadherin (KI E16P^+/+^) [28] at gestation day 15/19. Non-infected pregnant control mice were injected with PBS. After 72 h infection, as described previously [28], animals were killed with CO_2_ and aseptically dissected to isolate livers and placentas (6 to 12 placenta per pregnant mouse; atrophied placentas were not further used). Livers were kept on ice, while placentas were immediately frozen in liquid nitrogen and conserved at −80°C. Livers were disrupted with a tissue homogenizer in 3 ml of PBS and bacterial loads were determined by plating serial dilutions on BHI agar plates. Two independent experiments were performed with, respectively, 2 and 3 uninfected mice and 2 and 6 infected mice. Frozen placentas from the two mice that displayed the highest CFU numbers in liver lysates and from two uninfected mice were processed for RNA extraction and bacterial quantifications. Half of the placentas from each mouse were disrupted with a GentleMACS Dissociator (Miltenyi Biotec) in 1 mL RLT buffer supplemented with β-mercaptoethanol. RNA extraction from 350 µL of lysed organ was performed with RNeasy Mini Kit (Qiagen). The remaining half placentas were disrupted in 3 mL PBS with a tissue homogenizer and plated for CFU quantification on agar plates. ***(ii)*** *Study of* IFN-λ2 *responses in the placenta*. Pregnant B6.A2G-Mx1-IFNAR1^0/0^ mice [6] at gestation day 14, were treated subcutaneously with 5 µg of mouse IFN-λ2 (IL-28A; PeproTech) or PBS (mock) at 24 and 12 h prior to sacrifice. Embryos in utero were extracted, fixed with formaldehyde and embedded in paraffin. Tissues were sectioned and deparaffinized, followed by immunostaining for IFN-inducible Mx1 protein, as reported previously [6].

## Results

### *L. monocytogenes* Induction of IFN-III in LoVo Intestinal Epithelial Cells Requires the Invasion Proteins InlA and InlB and the Pore-forming Toxin LLO

**Figure 1:**
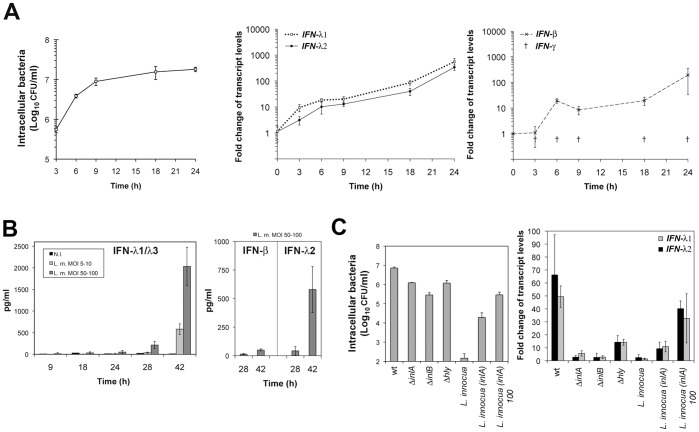
Characterization of *L. monocytogenes*-mediated induction of IFN-III genes in LoVo intestinal cells. A. LoVo intestinal epithelial cells infected with *L. monocytogenes* at multiplicity of infection (MOI) of 20 for the indicated time were lysed and processed for quantification of intracellular bacteria by counting colony-forming units (CFU) on agar plates, or for total cellular RNA extraction. Quantitative RT-PCR was performed to determine relative *IFN-λ1*, *IFN-λ2*, *IFN-β* and *IFN-γ* mRNA levels. The expression values were normalized to *GAPDH* transcript levels. Fold inductions were calculated from ΔΔCT values, using uninfected control cell values at the beginning of the experiment as a calibrator ( = 1). Values from three independent experiments are expressed as mean ± S.D. of the fold change. *IFN-γ* mRNA levels were below the limits of detection (†). At 24 h post-infection, fold change values in uninfected cells were 1.12±0.84 for *IFN-λ1*; 0.62±0.39 for *IFN-λ2*; 1.43±0.76 for *IFN-β*. **B.** Culture supernatants from LoVo cells infected with *L. monocytogenes* (L.m.), at the indicated time and MOI, were analyzed by ELISA for IFN production. Experiments were done in triplicate and reproduced once. **C**. LoVo cells were infected for 18 h with wild type (wt) or isogenic *ΔinlA*, *ΔinlB* or *Δhly L. monocytogenes* strains (MOI = 25), or with *L. innocua* or *L. innocua* expressing *inlA* (MOI = 25) or *L. innocua* expressing *inlA* (MOI = 100, indicated by “100”). Cells were processed as described in (A). Values are expressed as mean ± S.D of three independent experiments.

In an attempt to analyze the mechanisms underlying *L.* *monocytogenes* stimulation of IFN-III expression, we first determined bacterial loads and the kinetics of IFN gene expression over 24 h infection with *L. monocytogenes* in LoVo epithelial cells (Fig. 1A). *IFN-λ1* and *IFN-λ2* mRNA levels increased ∼10–20-fold over the first 6 h post-infection and then remained at this level until the 9 h time point. Subsequently, the mRNA levels rose again, reaching 40–80 fold at 18 h and up to 300–500 fold at 24 h. In contrast to this expression profile, *IFN-β* mRNA levels remained as in uninfected cells during the first three hours of infection. They then reached a plateau (∼20-fold induction) from 6 h to 18 h post-infection, and rose again up to ∼200-fold at 24 h. *IFN-γ* mRNA levels remained below the limits of detection of the assay, both in uninfected and infected LoVo cells, consistent with the fact that IFN-γ is produced primarily by immune cells.

We followed in parallel the production of IFN proteins in cell supernatants by ELISA assays. No IFN was detectable at any time point, except IFN-λ1/λ3 at 24 h (Fig. 1B and data not shown). Lysing the cells did not improve detection (data not shown). We thus performed longer infection kinetics and examined the effect of increasing the multiplicity of infection (MOI) on the production of IFNs. Secreted IFN-λ1/λ3 and IFN-λ2 were significantly detected in supernatants of infected cells at 28 h post-infection and their levels increased further at 42 h, reaching ∼2000 pg/ml for IFN-λ1/λ3 and ∼500 pg/ml for IFN-λ2, at a MOI of 50–100 (Fig. 1B). In contrast, the levels of secreted IFN-β remained very low, close to the detection limit of the ELISA assay, indicating that IFN-III are the major interferons produced and secreted by LoVo cells in response to *L. monocytogenes* infection.

The kinetics of IFN-λ expression suggested that there were two waves of gene activation (Fig. 1A) that might result initially from bacterial internalization and later from bacterial escape from the internalization vacuole and growth in the cytosol. To address this possibility, we compared the transcription of IFN-λ genes in LoVo cells infected for 18 h with wild type *L. monocytogenes* or isogenic Δ*inlA*, Δ*inlB* or Δ*hly* strains. *inlA* and *inlB* encode *L. monocytogenes* major invasion factors, which can both mediate listerial entry into LoVo cells [26], while *hly* encodes listeriolysin O (LLO), a pore-forming toxin that promotes bacterial escape into the host cytosol [29], [30]. As expected, mutant strains exhibited significantly reduced number of intracellular bacteria, when compared to wild type bacteria (Fig. 1C). Remarkably, expression of IFN genes in cells infected with Δ*inlA* or Δ*inlB* mutant strains were nearly as low as in uninfected cells (Fig. 1C). Induction of IFN-λ genes was also low in cells infected with *hly*-deficient bacteria, but not as much as for *inlA-* and *inlB*-deficient bacteria. The contribution of entry and vacuolar escape to *Listeria*-mediated induction of IFN-λ genes was further addressed by using the non-invasive species *Listeria innocua,* which does not produce InlA, InlB and LLO, and can be internalized into epithelial cells when expressing *inlA* [31], [32]. While *L. innocua* had no effect on the expression of IFN-λ genes, InlA-mediated internalization of *L. innocua* triggered their transcription, in a MOI-dependent manner (Fig. 1C). This result strongly suggets that *Listeria* internalization into a vacuole is sufficient to induce the expression of IFN-λ genes. However, induction levels always remained below that of *L. monocytogenes*, which can reach the cytosol.

Taken together, these results indicate that *L. monocytogenes*-mediated maximal induction of IFN-III in epithelial cells proceeds from both bacterial entry and escape into the host cell cytoplasm.

### Gram-positive Intracellular Bacteria Induce much more IFN-III than Gram-Negative Bacteria in LoVo Epithelial Cells

**Figure 2:**
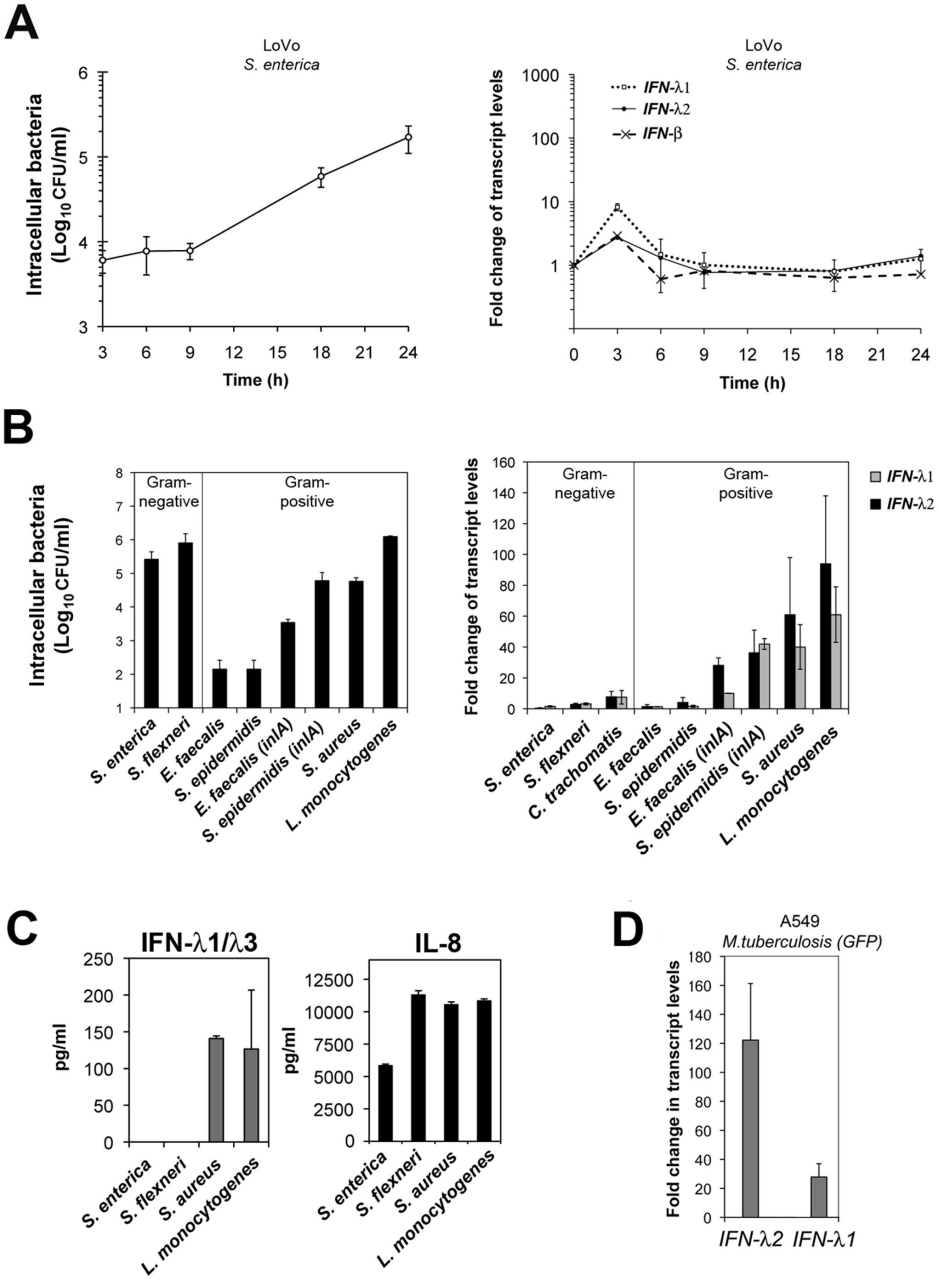
Induction of IFN-III genes by different bacterial species in epithelial cells. **A.** LoVo intestinal cells infected with *S. enterica* at multiplicity of infection (MOI) of 20 for the indicated time, were lysed and processed for quantification of intracellular bacterial by counting colony-forming units (CFU) on agar plates, or for total cellular RNA extraction and quantification of IFN gene expression, as described for *L. monocytogenes* in Figure 1A. Values from three independent experiments are expressed as mean ± S.D. of the fold change relative to uninfected control cell values at the beginning of the experiment. **B.** LoVo cells, uninfected or infected for 18 h with different bacterial species, were processed for quantification of bacterial load and mRNA. Quantification of internalized bacteria: data are means ± S.D. of CFU for 10^5^ cells (*n* = 3) at the indicated MOI, except for *C.* *trachomatis,* for which the percentage of infected cells (*n* = 4) were determined by flow cytometry after antibody labelling (see text). *IFN-λ1* and *IFN-λ2* transcript levels were determined by qRT-PCR and normalized to *GAPDH* transcript levels. Values are expressed as mean ± S.D. of the fold change relative to that in uninfected cells (*n* = 3 to 5). **C.** Quantification of IFN-λ1/λ3 and IL-8 production were done by ELISA, using culture supernatants of LoVo cells infected for 28 h with the indicated bacterium at a MOI of 50. Experiments were done in triplicates and reproduced once. **D**. Quantification of IFN-λ mRNAs in A549 lung epithelial cells infected with *GFP*-expressing *M. tuberculosis* (*n = 4*). The percentage of infected cells were determined by flow cytometry (see text).

We next studied whether other bacterial species could induce the expression of IFN-III genes in LoVo cells, starting with the Gram-negative *S. enterica sv.* Typhimurium, as this species is known to activate these genes in DCs [20]. Strikingly, IFN-λ and IFN-β gene expression profiles in *Salmonella*-infected cells (Fig. 2A) showed a markedly different pattern than in *Listeria*-infected cells (Fig. 1A). Transcript levels for both IFN slightly increased after 3 h of infection and returned to basal levels at 6 h in *Salmonella*-infected LoVo cells. This stimulation (∼10-fold for *IFN-λ1* and ∼3-fold for *IFN-λ2* and *IFN-β*) was considerably lower than that reported in DCs [20]. Moreover, although *Salmonella* efficiently replicated in LoVo cells, as seen by an increase in the number of intracellular bacteria over time, there was no further induction of IFN genes at later time points.

We then investigated the ability of other Gram-negative and Gram-positive bacteria to induce the expression of IFN-III genes. The numbers of intracellular bacteria were quantified in parallel by gentamicin assays (Fig. 2B), except for the obligate intracellular pathogen *Chlamydia trachomatis*, for which invasion efficiencies were determined by flow cytometry (47±8% of LoVo cells were positive for *C. trachomatis* infection). As shown in Fig. 2B, 18 h post-infection the levels of *IFN-λ1/λ2* mRNAs did not increase upon *Salmonella* infection, as described above (Fig. 2A), and increased by ∼3-fold and ∼ 8-fold upon *Shigella flexneri* or *C. trachomatis* infection, respectively, compared to the level of these transcripts in uninfected cells. These induction levels were far less than those observed for the Gram-positive bacterium *L. monocytogenes* (Fig. 1A, Fig. 2B). Like *L. innocua*, the non-invasive Gram-positive species *E.* *faecalis* and *S. epidermidis* failed to induce IFN-λ genes. However, InlA-mediated internalization of these bacteria induced *IFN-λ1* and *IFN-λ2* by ∼20–40-fold (Fig. 2B). We searched for another Gram-positive organism that could enter into LoVo cells without heterologous expression of *inlA*. Several studies showed that some strains of *Staphylococcus aureus* can be internalized in epithelial cells [33]–[35]. We found here that *S. aureus* SH1000 was significantly internalized into LoVo cells and, importantly, stimulated the expression of *IFN-λ1* and *IFN-λ2* transcripts by ∼40–60-fold (Fig. 2B). This result was confirmed at protein level. After 28 h of infection, amounts of IFN-λ1/λ3 in supernatants of cells infected with *S. aureus* were comparable to that in *L. monocytogenes*–infected cells (Fig. 2C). In contrast, there was neither IFN-λ1/λ3 nor IFN-λ2 in supernatants of cells infected with *S. enterica* or *S. flexneri* (Fig. 2C and data not shown). We noticed massive cell death in *Shigella*-infected cells after 42 h infection, but IFN-λs remained undectable in supernatants (data not shown). We checked that all of these bacterial species induced comparable levels of IL-8, a cytokine which is known to be induced by intracellular pathogens in epithelial cells [36], [37] (Fig. 2C). This indicates that LoVo cells can sense both Gram-positive and Gram-negative intracellular bacteria.

Altogether, these results highlight marked differences in the ability of bacterial species to specifically induce IFN-III in epithelial cells (Table 1) and suggest that Gram-positive bacteria are better inducers than Gram-negative bacteria.

### IFN-III Genes are Induced in A549 Lung Epithelial Cells in Response to *Mycobacterium Tuberculosis* Infection

*M. tuberculosis*, the causative agent of human tuberculosis, is not classified as either a Gram-positive or a Gram-negative bacterium. A key feature of this facultative intracellular pathogen is its ability to persist within human cells for long periods of time, especially in lung alveolar macrophages. However, it has also been reported to invade and replicate within alveolar epithelial type II pneumocytes *in vitro* [38]–[40] and also in the lungs of infected patients [41]. It was thus of interest to examine the effect of this major pathogen on IFN-III gene expression in a human lung epithelial cell line. GFP*-*expressing *M. tuberculosis* bacteria were incubated with A549 lung cells for 18 h. At that time, 41±1% of cells were positive for *M. tuberculosis,* as determined by flow cytometry, and *IFN-λ1* and *IFN-λ2* genes were induced by 25-fold and 120-fold, respectively (Fig. 2D). This result suggests that IFN-III genes might be produced in the lung epithelium during tuberculosis.

### IFN-III Genes are Induced in Epithelial Cells of Different Origins in Response to *L. monocytogenes* Infection

**Figure 3:**
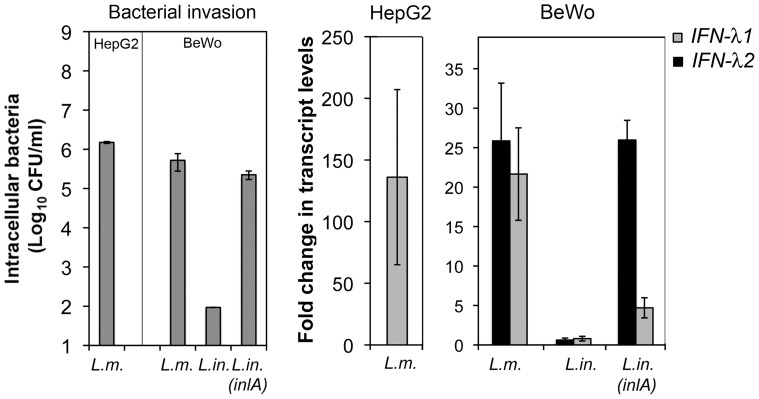
Induction of IFN-III genes by *L. monocytogenes* in HepG2 hepatocytes and BeWo trophoblastic cells. Quantification of bacterial loads (CFU) and IFN-λ mRNAs levels in *Listeria*-infected HepG2 or BeWo cells. *IFN-λ1* and *IFN-λ2* transcript levels were determined by qRT-PCR and normalized to *GAPDH* transcript levels. Values are expressed as mean ± S.D. of the fold change relative to that in uninfected cells (*n* = 3). *IFN-λ2* levels in uninfected HepG2 cells were below the detection threshold, preventing measures of fold change. *L. monocytogenes* (*L. m.*), *L. innocua* (*L. in.*), *L.* *innocua* expressing *inlA* (*L. in. (inlA)*).

During listeriosis, *L. monocytogenes* targets epithelial cells of the intestine, but also from other organs, such as the placenta and the liver. We found that *L. monocytogenes* and *inlA*-expressing *L. innocua* up-regulated IFN-III genes in human intestinal cells other than LoVo cells (Caco-2 cells, data not shown), as well as in BeWo trophoblastic cells (Fig. 3, Table 1). *L. monocytogenes* also highly induced *IFN-λ1* in HepG2 hepatocytes. In this cell line, *IFN-λ2* mRNA levels slightly increased upon infection, but the steady-state *IFN-λ2* levels in uninfected cells were below the detection limits, preventing quantitative measurement of a fold change. In contrast, *L. monocytogenes* infection had no effect on the expression of IFN-III genes in two non-epithelial human cell lines (HEK293 embryonic cells and THP-1 monocytes, data not shown). Thus, *L. monocytogenes* might specifically trigger the expression of IFN-III genes in its epithelial niches.

### IFN-III and IFN-responsive Genes are up-regulated During Mouse Fetoplacental Listeriosis

**Figure 4:**
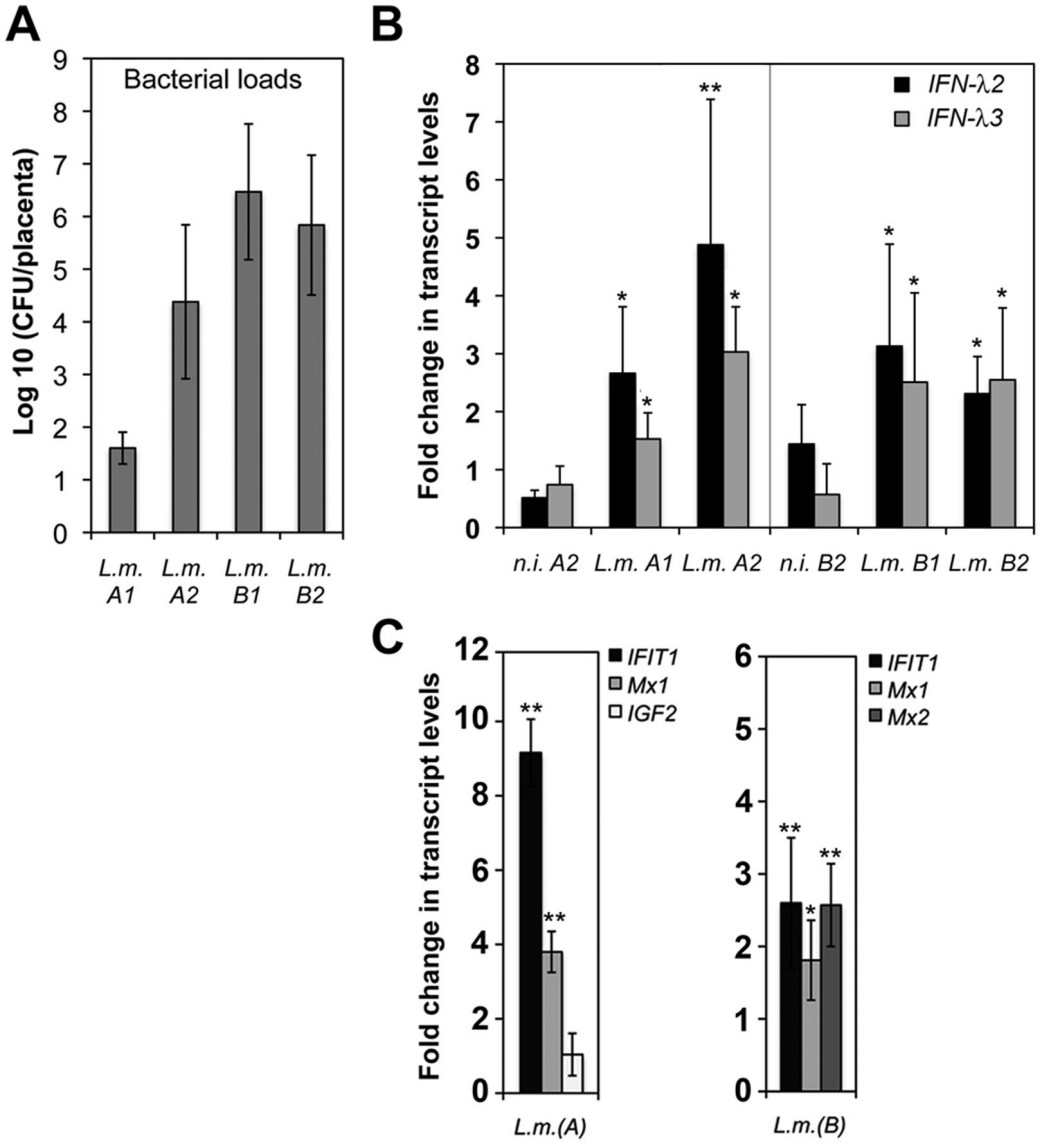
Induction of IFN-III and IFN-stimulated genes in mouse placenta. Pregnant E16P^+/+^ mice were inoculated intravenously with 4×10^4^ CFUs *L. monocytogenes* in PBS (*L.m.*) or with PBS only (*n.i.*) in two independent experiments (exp. A and B). Bacterial numbers were determined in livers and placentas 72 h post-infection. Placentas (*n* = 7 in exp.A; *n* = 8 in exp.B) from the two mice that displayed the highest bacterial loads in liver lysates, and placentas (*n* = 3) from two uninfected mice (*n.i.*), were processed for RNA extraction and bacterial quantifications. **A.** Quantification of *L. monocytogenes* loads and IFN-λ transcripts in placenta homogenates. **B.** Quantification of *IFN-λ2* and *IFN-λ3* transcript levels in placentas by qRT-PCR, with normalization to *GAPDH* transcripts. Values are expressed as mean ± S.D. of the fold change relative to that in placenta of uninfected mouse A1 or mouse B1 ( = 1). There is no significant change in uninfected mouse A2 or mouse B2 (*n.i. A2*, *n.i. B2*)**. C.** Quantification of transcript levels of IFN-responsive genes (*IFIT1, Mx1, Mx2)* and a control gene (*IGF2*) in placentas were determined by qRT-PCR and normalized to *YWHAZ* housekeeping gene. Values are expressed as mean ± S.D. of the fold change relative to that in placenta of all uninfected mice of exp.A or exp.B. (*p<0.05; **p<0.005, Student *t* test).

*L. monocytogenes* causes maternofetal infections in humans [42]. Since *L. monocytogenes* invades trophoblast cells *in vivo* [28] and induces IFN-III genes in these cells *in vitro,* we examined the possibility that IFN-λ might be induced upon infection at the placental barrier. A knock-in mouse line ubiquitously expressing humanized E-cadherin, the receptor for the *L. monocytogenes* invasion factor InlA, has been recently established for studying *L. monocytogenes* invasion of the placenta [28]. Bacterial loads, *IFN-λ2* and *IFN-λ3* transcript levels (murine *IFN-λ1* being a pseudogene) were quantified in placental homogenates 72 h after intravenous inoculation of pregnant mice with *L. monocytogenes* (Fig. 4A). We observed a 2-5-fold increase in the level of *IFN-λ2/λ3* mRNA in placentas extracted from infected pregnant mice; the increase was statistically significant when compared to uninfected controls (Fig. 4B). Similar results were obtained when the *YWHAZ* housekeeping gene, which has greater expression stability than *GAPDH* in placenta [43], was used for normalization instead of *GAPDH* (data not shown).

In an attempt to examine whether genes stimulated by IFN were induced in response to *Listeria* infection in the placenta, we quantified the expression of representative IFN-responsive genes, *IFIT1* and *Mx1/2,* in placenta infected or not by *L. monocytogenes.* As shown in Fig. 4C, 72 h after intravenous inoculation of mice with *L. monocytogenes,* the expression of *IFIT1* and *Mx1/2* was induced in placentas, in contrast to that of a control gene, *IGF2*. These results indicate that *L. monocytogenes* infection can promote IFN-λ expression and an IFN response in the placenta.

### IFN-λ2 Induces a Response in the Mouse Placenta

**Figure 5:**
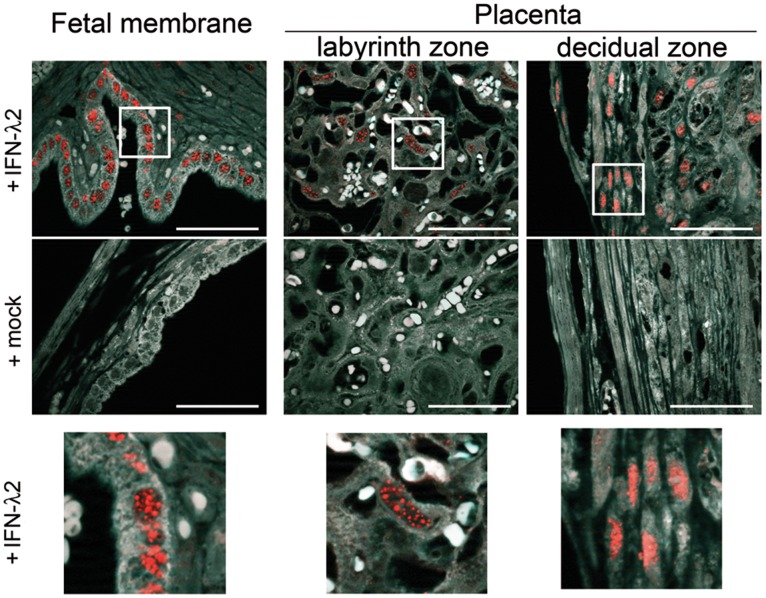
Response to IFN-III in mouse placenta and fetal membrane. Pregnant B6.A2G-Mx1-IFNAR1^0/0^ mice were treated subcutaneously with 5 µg of mouse IFN-λ2 or PBS (mock) at 24 and 12 h prior to sacrifice. Embryos *in utero* were extracted and fixed with formaldehyde. Sections of paraffin-embedded tissue were immuno-stained for IFN-inducible Mx1 protein. IFN-λ response was monitored by nuclear Mx1 staining (red) in epithelial cells of the fetal membrane and various regions of the placenta. Counterstaining: background auto-fluorescence (white). Zooms of squared regions are shown below. Scale bar: 50 µm.

Expression of IFN-responsive genes in infected placentas might not only be due to IFN-III, as IFN-I induces similar set of genes [11]. In fact, it is unknown whether the placenta can repond to an IFN-III stimulus. To determine whether placental epithelial cells could specifically respond to IFN-III *in vivo*, we used a reporter mouse line that lacks IFN-I receptors (IFNAR1^0/0^ mice) and carries a functional *Mx1* allele [4]–[6]. Staining for Mx1 in IFNAR1^0/0^ mice represents a powerful tool to identify cells that specifically respond to IFN-III, as Mx1 is an IFN-induced protein that rapidly accumulates in the nucleus of responsive cells [4]–[6]. Since these mice do not express the humanized E-cadherin receptor, *L. monocytogenes* could not be used as a stimulus for the IFN-III pathway in placental epithelial tissues of these mice. Instead, we used purified recombinant IFN-λ2 and observed that subcutaneous treatment with this cytokine induced prominent nuclear Mx1 staining in epithelial cells of the fetal membranes, as well as decidual and labyrinth zones of the placenta (Fig. 5). This result demonstrates that the fetoplacental tissue is highly responsive to IFN-III.

## Discussion

In this study, we show that IFN-III are the major interferons produced and secreted by epithelial cells in response to *L.* *monocytogenes* infection, by mechanisms involving both the internalization and cytosolic phases of infection. We report that other major bacterial pathogens, *M. tuberculosis* and the Gram-positive cocci *S. aureus*, also stimulate type III IFN expression in epithelial cells. In contrast, Gram-negative pathogens *S. enterica*, *S.* *flexneri* and *C. trachomatis* have no or only weak effects. We also provide evidence for *in vivo* induction of these genes during infection of placental tissue by *L. monocytogenes*. IFN-I and IFN-III genes can potentially be expressed by all nucleated cells, following the activation of pattern recognition receptors (PRR) by microbial products [21], [22]. Most studies have nevertheless focused on the expression of these genes in immune cells or fibroblasts. The results presented here highlight that there are marked and intriguing differences between bacterial species in their ability to activate IFN-III genes in epithelial cells, and this could play an important role in epithelial immunobiology.

Among different bacteria tested, *L. monocytogenes* highly induces *IFN-λ1/λ2* transcription in intestinal cells and other cells of epithelial origin, such as cytotrophoblast and hepatocytes, extending our previous findings [23]. Induction of IFN-III genes by *L. monocytogenes* in epithelial LoVo cells occurs in two waves, the first probably involving InlA- and InlB-mediated internalization, and the second involving cellular events promoted by LLO-mediated vacuolar escape. Based on these observations, we hypothesize that both vacuolar and cytosolic immune surveillance pathways contribute to IFN-III production. Indeed, it has been shown that *L. monocytogenes* infection induces distinct immune responses in macrophages, depending on whether it acts on the plasma membrane, the vacuole or the cytosol [44], [45]. However, LLO-deficient bacteria fail to induce type I IFN in these immune cells, suggesting that only the cytosolic surveillance pathway is responsible for *L. monocytogenes*-induced IFN-I [46]. Here, we found that while internalization contributes to IFN-λ induction in epithelial cells, LLO-deficient bacteria are only partially defective in eliciting this response. Moreover, *IFN-λ1/λ2* genes start to be expressed earlier than *IFN-β* upon infection, and IFN-λs are produced at higher level than IFN-β. These differences suggest that IFN-III induction during epithelial cell infection may not use the same mechanisms than those leading to IFN-I expression in macrophages [18], [21], [45], [47]. In this respect, *Listeria* could be a useful tool to investigate such specificities.

We highlight that other Gram-positive bacteria also significantly induce the expression of IFN-III genes upon internalization in epithelial cells. In particular, two major pathogens, *S. aureus* and *M. tuberculosis*, induce IFN-λ in LoVo intestinal and A549 lung epithelial cells, respectively. Both species lead to chronic infections in humans, and an emerging body of evidence suggests that they can reside as intracellular pathogens in epithelial cells [33]–[35], [38]–[40], which would constitute a reservoir involved in bacterial persistence *in vivo* [41], [48]–[50]. Interestingly, IFN-III was recently described as a modulator of the T-helper 2 (Th2) response, with inhibitory effects on Th2 cell-mediated inflammation [51], [52], as well as a suppressor of allergy in the lung [53]. Therefore, it is possible that continuous induction of IFN-III by epithelial cells in mucosa may strategically help persistence of these pathogens.

In contrast, Gram-negative *S. flexneri*, *S. enterica* and *C. trachomatis* species do not or only weakly induce IFN-λ genes in LoVo cells, suggesting that Gram-negative and Gram-positive organisms might differentially target PRR signaling cascades leading to IFN-III production in epithelial cells. Such differences in the induction of immune response genes between different groups of bacteria have been reported in other cells types; for instance, Gram-positive and Gram-negative bacteria induce different patterns of pro-inflammatory cytokines in human monocytes [54]. The differences observed do not seem to result from different amounts of intracellular bacteria (Fig. 2B) or from localization in different cellular compartments. *L. monocytogenes* and *S. flexneri* replicate in the cytosol with similar efficiency, yet only *L.* *monocytogenes* stimulates IFN-III production. The differences between Gram-positive and Gram-negative bacteria might result from production of distinct MAMPs and/or factors influencing IFN signaling pathways, or from epithelial cell specificities in the repertoire of PRRs. In fact, LPS that is produced by both *Shigella* and *Salmonella* activates IFN-λ genes in other cell types [19], [20] and *S. enterica* itself induces IFN-λ in DCs [20]. However, as shown here, these enteropathogens do not trigger expression of IFN-λ genes in LoVo intestinal cells. They might use specific mechanisms to actively dampen IFN-III expression in this cell type.

So far, the function of IFN-III in bacterial infection is unknown. From viral infection studies [4]–[6] and owing to the receptor restricted expression pattern, it is tempting to speculate that IFN-λ contributes to epithelial innate immunity in response to bacteria, but not necessarily for the host benefit. Indeed, while type II IFN (IFN-γ) has antibacterial activity, type I IFN favors *L. monocytogenes* and *M. tuberculosis* persistence [21], [22]. A first step before understanding the role of IFN-III in bacterial infectious diseases, in particular listeriosis, was to find out whether these genes are induced in epithelial tissues *in vivo*. To address this question, we used a mouse model in which *L. monocytogenes* can efficiently invade epithelial cells due to the expression of its humanized receptor E-cadherin [28]. *Listeria* colonizes several tissues of epithelial origins such as the the liver, intestine and placenta. We chose to study the expression of IFN-III in the murine placenta for three reasons: (*i*) the placenta has not yet been described as an IFN-λ-producing or -responsive tissue; (*ii*) IFN-λ elicits a response in the mouse intestine [55], but this tissue is in contact with the numerous bacteria of the microbiota, which may affect IFN production; (*iii*) IFN-λ receptor is expressed at very low levels in mouse liver, in contrast to human liver, and thus IFN-λ has no effect in this organ in the mouse model [4], [55], [56]. We report that levels of *IFN-λ2* and *IFN-λ3* mRNAs and that of the IFN-responsive genes *IFIT1* and *Mx1/2* are increased in the placenta infected with *L. monocytogenes*, indicating that IFN-III may participate in the immune response at the fetoplacental barrier. Supporting this hypothesis, cells of the fetal membranes and decidual and labyrinth zones of the mouse placenta respond to IFN-λ2 treatment (Fig. 5).

Whether IFN-λ could mediate protection of the fetus from invading *Listeria,* or alternatively, whether this pathway is beneficial for the pathogen, for instance by stimulating abortion that leads to bacterial release in the environment, deserves future investigations. In this regard, in-depth analysis of infection kinetics and the establishment of new animal models are required, in particular generation of a mouse line that would be both permissive for *Listeria* infection of epithelia and impaired in IFN-III responses. However, one should keep in mind that the mouse model may not be optimal to address the role of IFN-III in human listeriosis, since *IFN-λ1* is a pseudogene in mice, while human cells produce this cytokine upon infection with *L. monocytogenes*.

Most pathogenic bacteria target tissues of epithelial origin, such as skin, throat, gut, liver, lung, genital mucosa or placenta. We propose that some bacterial species allow epithelial cells to become a source of IFN-λs, acting as paracrine immunomodulators of mucosal surfaces. Dissecting the mechanisms of IFN-III production and function during bacterial diseases may have important implications for diagnostic and therapeutic developments.
